# Refining the in vitro release test method for a dapivirine-releasing vaginal ring to match in vivo performance

**DOI:** 10.1007/s13346-021-01081-7

**Published:** 2021-10-21

**Authors:** Diarmaid J. Murphy, Deanna Lim, Ryan Armstrong, Clare F. McCoy, Yahya H. Dallal Bashi, Peter Boyd, Tiffany Derrick, Patrick Spence, Bríd Devlin, R. Karl Malcolm

**Affiliations:** 1 School of Pharmacy, Queen’s University Belfast, Belfast, BT9 7BL UK; 2 International Partnership for Microbicides, Silver Spring, MD USA

**Keywords:** Vaginal ring, In vitro release testing, Biorelevant, Drug release, Dapivirine

## Abstract

Previously reported in vitro release test methods for drug-releasing vaginal rings containing poorly water-soluble drugs have described use of water-alcohol systems or surfactant solutions in efforts to maintain sink conditions. Here, as part of efforts to more closely match in vitro and in vivo release for the 25 mg dapivirine matrix-type silicone elastomer vaginal ring for HIV prevention, we have investigated alternatives to the 1:1 v/v water/isopropanol medium described previously. Specifically, we evaluated dapivirine release from rings into (i) monophasic water/isopropanol mixtures of varying compositions and (ii) biphasic buffer/octanol systems using pH 4.2 and pH 7.0 buffers. The rate and mechanism of dapivirine release were dependent upon the isopropanol concentration in the release medium, in accordance with the observed trend in drug solubility. At 0 and 10% v/v isopropanol concentrations, dapivirine release followed a partition-controlled mechansim. For media containing ≥ 20% v/v isopropanol, in vitro release of dapivirine was significantly increased and obeyed permeation-controlled kinetics. Cumulative release of ~3.5 mg dapivirine over 28 days was obtained using a water isopropanol mixture containing 20% v/v isopropanol, similar to the ~4 mg dapivirine released in vivo. Dapivirine release into the biphasic buffer/octanol system (intended to mimic the fluid/tissue environment in vivo) was constrained by the limited solubility of dapivirine in the buffer component in which the ring resided, such that cumulative dapivirine release was consistently lower than that observed with the 20% v/v isopropanol in water medium. Release into the biphasic system was also pH dependent, in line with dapivirine’s pK_a_ and with potential implications for in vivo release and absorption in women with elevated vaginal pH.

**Graphical abstract:**

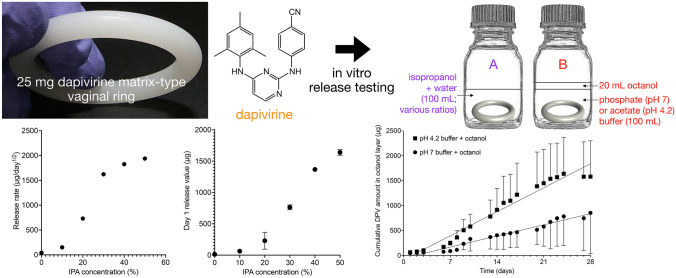

**Supplementary information:**

The online version contains supplementary material available at 10.1007/s13346-021-01081-7.

## Introduction

Methods to measure in vitro release of drug substances–commonly referred to as ‘in vitro release testing (IVRT)’ or ‘dissolution testing’–are a regulatory requirement for many new drug products [1–6]. For sustained/controlled release dosage forms such as vaginal rings, methods typically involve sampling at multiple time points extending over the same time period as intended for clinical use [4, 5, 7]. In early-stage product development, IVRT can be useful in differentiating between formulations and helping determine critical material attributes, process parameters, and quality attributes of the intended product [8]. In the later stages of development, IVRT is used to set specifications for batch release, determine batch-to-batch variability, and potentially investigate product differences through changes in formulation or manufacturing [8].

The distinctions between quality control (QC) dissolution, biorelevant dissolution, and clinically relevant dissolution methods have been described previously [8, 9]. Ideally, all three make use of the same method. In practice, however, they usually do not. The QC test is expected to be less complex and more robust, while maintaining the ability to detect meaningful variations in product performance [9]. Regulators are keen that in vitro test results are linked with in vivo product performance to make dissolution testing ‘clinically relevant’ [8, 10–12]. At the same time, concerns have been raised about the use of more complicated, biorelevant in vitro release tests for routine quality control purposes [8, 13].

The development of simple, practical, and robust in vitro release test methods for drug-releasing vaginal rings needs to account for the specific drug/formulation characteristics and the intended clinical use of these products. Important factors to consider may include the following: the poor water solubility of many drugs formulated in vaginal rings; the relatively large initial drug loadings; the relatively low drug release rates; the extended time periods over which drug release is required (typically ranging from 3 weeks to 12 months); and the often significant quantities of residual drugs remaining in the device after clinical use [4, 5, 14, 15]. For maintenance of sink conditions, it is common practice to use either relatively large volumes (e.g. 100–400 mL) of release medium–sampled and replaced regularly, e.g. daily or twice weekly–or lesser volumes of release medium containing a water-miscible solvent or surfactant to enhance drug solubility and therefore maintain permeation-controlled (also referred to as *diffusion-controlled*) drug release kinetics [4, 16–19]. A small number of previous studies have investigated drug release from vaginal rings under both sink and non-sink conditions [20, 21], since sink conditions may not be operative in vivo. However, in general, the majority of IVRT methods for vaginal rings maintain sink conditions using either water-solvent systems or aqueous surfactant solutions. This is not without precedent–the FDA dissolution database describes many surfactant-based methods for testing drug products containing poorly water-soluble drugs, and a couple of methods describe use water-alcohol mixtures (including methanol and isopropanol) [22].

Many factors can potentially influence the release and absorption of drugs following vaginal administration in humans, including location of placement of the dosage form, vaginal microflora, the composition and volume of vaginal fluid, vaginal pH, the stage of menstrual cycle, age-related change in the thickness and laxity of vaginal epithelial tissue, and vaginal infections [23–30]. Various vaginal fluid simulants useful for in vitro release testing of vaginal drug products have been described in the literature [24, 30, 31]. However, a simple pH 4.2 simulated vaginal fluid (SVF) [24] is widely used, and many commercial vaginal rings make use of simple phosphate-buffered saline [4, 15, 16, 18, 32, 33]. Given the obvious difficulties in attempting to replicate the complex vaginal system in an in vitro release test, most published methods have prioritised pH control and maintenance of sink conditions rather than attempt to more closely match biological and physiological conditions.

A vaginal ring containing 25 mg of the antiretroviral drug dapivirine (DPV) has recently received a positive scientific opinion from the European Medicines Agency (EMA) for use by women in developing countries and a recommendation from the World Health Organization as an additional HIV prevention option for women. The product is currently under regulatory review in eastern and southern Africa and the USA. Its efficacy in reducing the incidence of HIV acquisition has been demonstrated in two phase III clinical trials [34, 35]. Previous studies have reported the use of two different IVRT methods for this device: a 1:1 v/v mixture of isopropanol/water (IPA/water) and a solution of SVF containing the surfactant Tween 80 (SVF/Tween) [16, 17]. The IPA/water mixture is very simple to prepare but overestimates the total amount of DPV released over 28 days in vivo. For example, release of ~13 mg over 28 days into IPA/water was measured compared to the ~4 mg released in vivo [16, 36]. Although SVF/Tween is more biorelevant, it requires longer preparation times and a relatively large quantity of surfactant (0.2% w/v) to match the total amount of DPV release measured in vivo. Here, we have investigated in vitro release of DPV using alternative media–monophasic IPA/water mixtures having different compositions and biphasic aqueous buffer/octanol systems having different buffer pH–with the aim of identifying a release medium that, without added surfactant, more closely matches cumulative DPV release in vivo. Biphasic media have previously been used for dissolution testing of solid dosage forms [37]. To the best of our knowledge, this is the first time they have been formally investigated for drug release testing of vaginal rings.

## Materials and methods

### Materials

 Matrix-type silicone elastomer vaginal rings containing 25 mg DPV (Ring-004, mean measured DPV content per ring 24.4 ± 0.15 mg) were manufactured at Sever Pharma Solutions (formerly QPharma; Malmö, Sweden) and supplied by the ring’s developer and regulatory sponsor, the International Partnership for Microbicides (IPM). Potassium dihydrogen orthophosphate, acetic acid, and potassium hydroxide (AnalaR, analytical reagent grade) were purchased from VWR International Ltd. (Dublin, Ireland). HPLC-grade isopropanol (IPA), acetone, acetonitrile, phosphoric acid (85% w/w in water), sodium dihydrogen phosphate, hydrochloric acid (0.5 M), potassium hydroxide (concentrate) and potassium hydrogen phthalate, and 1-octanol were all purchased from Sigma-Aldrich (Gillingham, UK). Phosphate buffer (pH 7) for use in the Sirius instrument was supplied by Thermo Scientific (Loughborough, UK). A Millipore Direct-Q 3 UV Ultrapure Water System (Watford, UK) was used to obtain HPLC-grade water. Different compositions of IPA/water (0/100, 10/90, 20/80, 30/70, 40/60, and 50/50 v/v) were prepared. Phosphate buffer (0.009 M, pH 7.0) and acetate buffer (0.001 M, pH 4.2) were prepared fresh before use. Syringe filters (4 mm) containing 0.2 µm PTFE membranes were from Phenomenex (Cheshire, UK) and polypropylene tubes (15 mL) from VWR (Dublin, Ireland).

### Determination of dapivirine physicochemical characteristics

The ionisation constant (pK_a_) for dapivirine was measured by titration using a Sirius T3 (Pion Inc, UK). The apparent ionisation constant was determined in a mixed solvent mixture comprising 20% acetonitrile, 20% dioxane, and 20% methanol (v/v) in 0.15 M KCl. Yasuda-Shedlovsky extrapolation of the measured values to 0% co-solvent was used to determine the aqueous pK_a_ value [38, 39]. Solubility of dapivirine in the various solvent systems was determined by adding solid micronised dapivirine to polypropylene centrifuge tubes (~15–20 mg for each ratio of IPA/water, 5–10 mg for the phosphate and acetate buffer samples, and ~0 mg for the octanol samples) before 10 mL of each test medium was added. The tubes were sealed and vortex mixed for 20 s before being placed in an orbital shaking incubator at 37 °C, 60 rpm for 72 h. Bottles were then placed in a cupboard at room temperature to equilibrate before being centrifuged at 7500 rpm for 3 min (Eppendorf 5430 R, Mason Technology, Ireland). The octanol and 50/50–20/80 IPA/water samples were subsequently diluted in ACN:water for analysis. The phosphate, acetate, and unbuffered water samples as well as the 10/90 (IPA/water) samples showed poor powder wetting, and centrifugation did not remove surface solid in these solvents. These samples were subsequently filtered through a 0.2-µm PTFE membrane filter prior to dilution and analysis. The membrane was initially flushed with an intermediate polarity solvent before being flushed with the solvent in question, and ≥ 2 mL of saturated solution was subsequently filtered through the membrane before the sample for analysis was taken to saturate the membrane with the solute. Each solvent system was investigated in triplicate.

### In vitro release testing of rings using various ratios of IPA/water

**Fig. 1:**
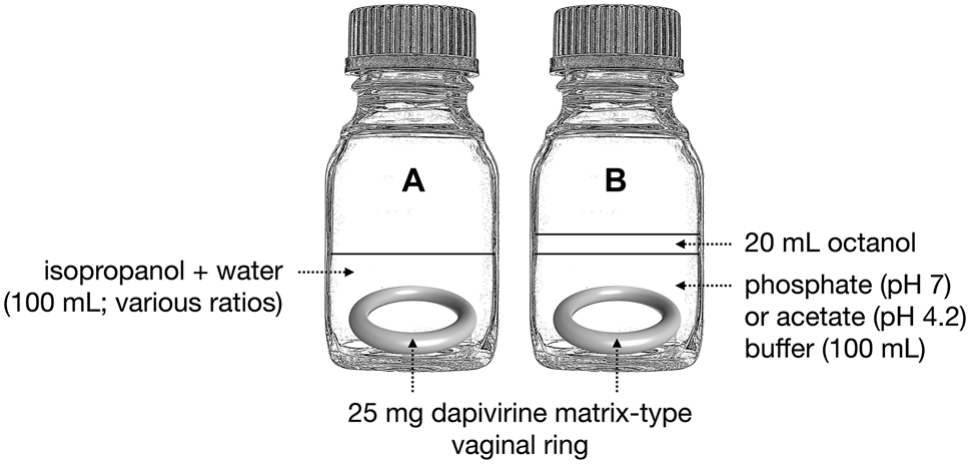
Flask setup for in vitro release testing methods. Vaginal ring placed into **A** 100 mL monophasic isopropanol + water mixtures, or **B** biphasic system comprising 100 mL buffer and 20 mL octanol

Rings were weighed and placed in individually labelled 250-mL glass flasks prior to addition of 200 mL of release medium containing water and 0 to 50% IPA in 10% increments (Fig. 1A). Four rings were tested for each release medium composition. Flasks were sealed and placed in an orbital shaking incubator at 37 °C, 60 rpm. After 24 h, flasks were removed and 1–2 mL of the medium was sampled directly into HPLC vials. The remainder of the medium was discarded and replaced with 100 mL fresh medium containing the appropriate ratio of IPA/water. Subsequent samples were taken daily, and the unsampled media discarded and replaced with 100 mL of fresh medium. No samples were taken over weekends; instead, 200 mL release medium was used each Friday to maintain equivalent release rates over the weekend.

### In vitro release testing of rings using a two-phase buffer/octanol system

Rings were weighed and placed in individually labelled 250-mL glass flasks prior to addition of 100 mL of either phosphate buffer (pH 7) or acetate buffer (pH 4.2). 1-Octanol (20 mL) was then slowly added to form a layer on top of the buffer solution (Fig. 1B) ensuring that the octanol layer did contact directly with the ring, before sealing the flasks and transferring them to an orbital shaking incubator at 37 °C, 60 rpm. After 24 h, flasks were removed from the incubator, the aqueous and octanol phases sampled separately (1.0 mL), and replaced with fresh medium (1 mL). Sampling of the aqueous phase was performed with either micropipettes or a needle and syringe (from day 14). The octanol phase was sampled with a positive displacement pipette for accurate solvent sampling and dispensing. Care was taken throughout addition and sampling of octanol to prevent the octanol layer from making direct contact with the ring. After sampling, flasks were returned to the incubator. Aqueous samples were analysed directly, while octanol samples were diluted 1:20 in HPLC mobile phase prior to HPLC analysis.

### HPLC method

A Waters HPLC system (Elstree, UK) consisting of a 1525 binary HPLC pump, a 717 plus autosampler, an in-line degasser unit, a 1500 series column heater, a 2487 dual wavelength absorbance detector, and a 2998 photodiode array detector was used for all analyses. Release and solubility samples (10 µL), appropriately diluted, were injected onto a Thermo Scientific BDS Hypersil™ C18 HPLC column (150 mm × 4.6 mm, 3 µm particle size) maintained at 25 °C and fitted with a guard column. Isocratic elution was performed using a mobile phase comprising 60% HPLC-grade acetonitrile and 40% phosphate buffer (pH 3.0) and a total flow rate of 1.5 mL/min. The run time was 6 min and DPV was detected at 287 nm after ~2.85 min. DPV amounts were determined using a weighted calibration curve generated from three separate sets of dilutions performed independently in IPA/water (Supplementary Information, Fig. S1 and Table S1).

### Analysis of release data

For the IPA/water method, release data were used to construct mean daily and cumulative release versus time (or root time) plots and mean dapivirine release rates determined by linear regression analysis [40, 41]. Release profiles were also assessed using the Peppas equation with log fractional release plotted against log time allowing calculation of the values of *k* and *n*, again using linear regression [42, 43]. For the biphasic release method, cumulative amounts of DPV in buffer and octanol were calculated from the daily release samples, accounting for the small amount removed in daily sampling.

## Results and discussion

### Physicochemical characterisation of dapivirine

Measured physicochemical and solubility data for dapivirine are presented in Table 1. Dapivirine contains a weakly basic diaminopyrimidine chemical functional group. pK_a_ and log *P* measurements (5.30 and 5.35, respectively) were determined by titration in a mixed solvent system (acetonitrile, dioxane, and methanol in potassium chloride) and extrapolated to zero co-solvent to derive the aqueous value using the methods of Yasuda-Shedlovsky (*R*^2^ = 0.9892, Supplementary Information, Fig. S2). The determined values are similar to, but slightly lower than, previously published values of 5.8 and 6.3, respectively [44]. The solubility values for DPV increase with degree of ionisation, in line with expectation [45, 46]. Also, addition of increasing amounts of IPA leads to a dramatic increase in the measured solubility [47].

**Table 1:** Chemical structure and some determined physicochemical properties of dapivirine including solubility in a range of buffers, IPA/water mixtures, octanol, and silicone elastomer

Chemical structure	Selected physicochemical properties	Solubility in various media (µg/mL); *(mg/g)	Solubility in IPA/water mixtures (µg/mL)
	MW: 329.4 g/mol	Unbuffered water: 0.084 ± 0.021	10/90: 0.556 ± 0.034
	pK_a_: 5.30 ± 0.02	Phosphate buffer pH 7: 0.018 ± 0.001	20/80: 2.76 ± 1.07
	log *P*: 5.35 ± 0.08	Acetate buffer pH 4.2: 0.499 ± 0.003	30/70: 24.14 ± 1.76
		Octanol: 6058 ± 116	40/60: 178.6 ± 11.6
		Silicone elastomer: 0.34*	50/50: 645.0 ± 5.2

*MW* molecular weight, *IPA* isopropyl alcohol. *[49]

### In vitro release testing of rings using various ratios of IPA/water

**Fig. 2:**
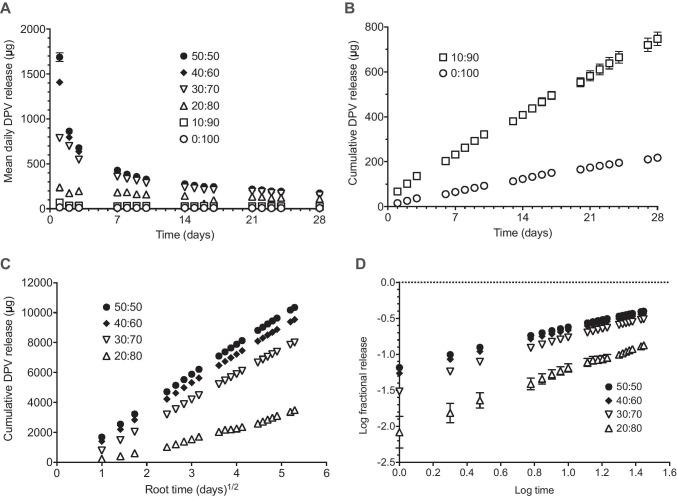
**A** Mean daily release of dapivirine from 25 mg vaginal rings into different release media composed of various ratios of IPA/water (0–50% IPA). **B** Mean cumulative release plotted against time for the 0/100 and 10/90 IPA/water mixtures. **C** Mean cumulative release plotted against root time for the 20/80 to 50/50 IPA/water mixtures. **D** Log fractional release plotted against log time for the 20/80 to 50/50 IPA/water mixtures. Each data point is the mean of four replicates and the error bars indicate the standard deviation of the measurement in each case; errors bars are often smaller than the plot symbols

Plots of mean daily DPV release versus time for 25 mg DPV rings in the various IPA/water media show that DPV release decreases with decreasing % IPA (Fig. 2A). For example, 190.4 µg DPV is released on day 24 in 50/50 IPA/water compared to just 5.7 µg in water only. Also, the drug burst commonly observed during the first few days with matrix-type rings [4, 15, 17, 20] is significantly reduced (relative to later release values) with use of media containing lower IPA concentrations (0–20%). This is due to the limited solubility of the highly lipophilic DPV in these predominantly aqueous media. For the water-only and 10/90 IPA/water media, linear correlations were obtained for the cumulative DPV release versus time plots (Fig. 2B). For release media containing ≥ 20% IPA, mean cumulative DPV release versus root time plots were linear (Fig. 2C). These plots helpfully illustrate the substantial increases in cumulative DPV release with increasing IPA from 0 to 30%, and the relatively smaller (but still significant) changes with further increases in IPA concentration (30–50%). A small dip was observed in both the daily and cumulative release profiles for the 20/80 IPA/water medium from days 14 to 16, and was attributed to an error in preparing the IPA/water mixture.

The log fraction released versus log time data (following the Peppas equation) are plotted in Fig. 2D [42, 43]. Summary DPV release data derived from the cumulative release plots–plotted either as cumulative release versus time or root time (Higuchi model), or as the log fractional release versus log time (Peppas model)–are presented in Table 2. The data demonstrate how increasing the concentration of IPA in the release media impacts the rate of DPV release from the ring. Increasing the IPA concentration from 0 to 10% and from 10 to 20% leads to a nearly fourfold increase in release rate, and there is a further doubling in release rate from 20 to 30% IPA. Subsequent increases in the IPA proportion produce smaller increases in release rate. This suggests that the transition from partition-controlled to matrix-controlled release occurs between the 10 and 20% IPA concentrations. However, as can be seen by the continued increase in release rates with increasing IPA proportions above 20%, the transition is quite broad. Similarly, analysis using the Peppas model shows that as the IPA content of the medium increases, the value of *n* decreases towards true Fickian release, e.g. for 40/60 and 50/50 IPA/water medium, the value of *n* was calculated as 0.562 and 0.535 respectively. As with the Higuchi model, there is a shift away from diffusion-controlled release as the IPA concentration in the release medium falls and DPV solubility plays a larger role. Based on the mean concentrations determined in the daily release samples, and the measured solubilities presented in Table 1, sink conditions are present throughout the 28 days (defined as less than 10% saturation solubility) [48], in the 50/50 and 40/60 IPA/water mixtures. Using a more relaxed definition of sink conditions (defined as less than 30% saturation solubility) would also include the 30/70 IPA/water mixture. The values determined for the 20/80 IPA/water medium should be interpreted with caution in relation to the Peppas model as the daily release values are typically greater than 40% of the saturation solubility of DPV in the medium, suggesting that sink conditions may not be present. As the 0 and 10% IPA containing media clearly do not represent sink conditions, the results for these media are not presented.

**Table 2:** Mean cumulative amount released on day 28, cumulative release rate equation gradients and intercepts with 95% confidence intervals in brackets, and coefficients of determination for DPV release into various proportions of IPA/water using the Higuchi and Peppas models

Release medium (% IPA/water)	Mean cumulative amount released on day 28 (mg) ± SD	Higuchi model			Peppas model		
		Slope (µg/day^1/2^ or *µg/day)	Intercept	*R*^2^ value	Slope (*n*)	Intercept (*k*)	*R*^2^ value
0/100^*^	0.218 ± 0.004	7.55 (7.41, 7.69)	14.24 (11.9, 16.58)	0.9931	N/A	N/A	N/A
10/90^*^	0.75 ± 0.03	25.0 (24.51, 25.51)	58.48 (50.25, 66.70)	0.9922	N/A	N/A	N/A
20/80	3.49 ± 0.31	766.1 (723.8, 808.4)	−723.4 (−883.4, −563.5)	0.9427	0.817 (0.7751, 0.8589)	−2.044 (−2.090, −1.998)	0.9507
30/70	8.01 ± 0.07	1693 (1685, 1702)	−903 (−935.9, −870)	0.9995	0.6638 (0.6505, 0.6771)	−1.447 (−1.462, −1.433)	0.9922
40/60	9.54 ± 0.20	1904 (1879, 1929)	−430.5 (−524.9, −336.2)	0.9966	0.5622 (0.5547, 0.5698)	−1.235 (−1.244, −1.227)	0.9965
50/50	10.35 ± 0.11	2022 (2004, 2040)	−238.5 (−306.7, −170.2)	0.9984	0.535 (0.5294, 0.5406)	−1.163 (−1.169, −1.157)	0.9978

^*^Partition-controlled release, cumulative release was plotted against time; not evaluated using the Peppas model as sink conditions are not present

The average cumulative amount of DPV released over 28 days clinical use of a 25 mg ring is approximately 4 mg [36]. This was most closely matched with the 20/80 IPA/water media for which cumulative release was 3.5 mg over 28 days. This medium falls within the intermediate zone where release is transitioning from diffusion controlled to solubility controlled. Note that the value of the *R*^2^ correlation coefficient for this data set plotted against time was 0.9348, which is lower than the 0.9427 calculated when the data were plotted against root time. The root time *R*^2^ value is also reduced by the unexpected dip in release observed between days 14 and 16 in the data with this release medium. Using the release rate equation to back-calculate the expected release without this dip gives an increase in overall cumulative release of approximately 0.12 mg.

**Fig. 3:**
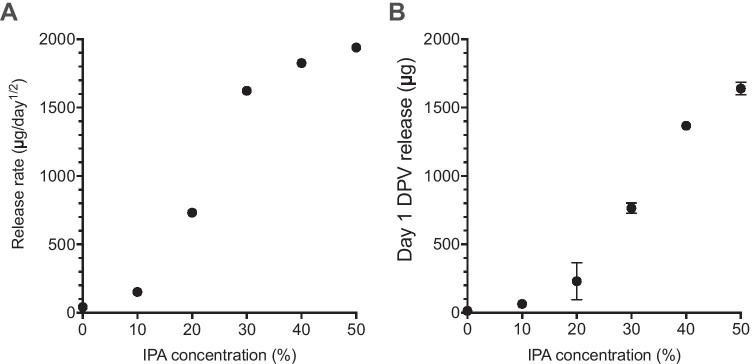
**A **Mean calculated release rate and **B** mean day 1 burst release value plotted against the proportion of IPA present in the release medium. Errors bars representing standard deviations (*n* = 4) are plotted, but are often smaller than plot symbols

A plot of the release rate calculated against the proportion of IPA present is displayed in Fig. 3A; in the plot, all release rates are calculated with respect to root time for consistency, despite this being less appropriate for the 0 and 10% IPA release media. This demonstrates the dramatic increase in release initially observed upon addition of IPA, followed by a relatively smaller but still significant increase observed as the proportion of IPA increases beyond 30%. Subsequent changes may reflect increasing solubility. The continued increase in release rate with increasing IPA up to 50% suggests that, as a minimum, the transition phase falls somewhere within 20–40% IPA range [50]. Figure 3B presents a plot of the day 1 release value (often described as the burst release value) for rings as the percentage of IPA in the release medium is increased. This data demonstrates the large impact that solubility plays on increasing the day 1 release.

### In vitro release testing of rings using a two-phase buffer/octanol system

**Fig. 4:**
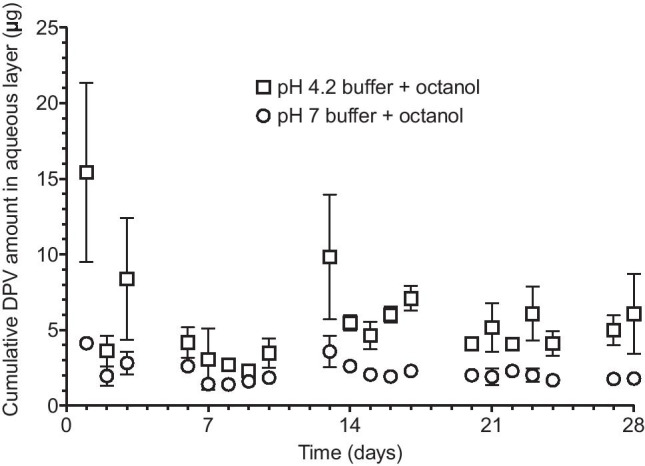
Mean cumulative amount of DPV measured in the aqueous buffer phase of the buffer/octanol system for phosphate buffer (pH 7) + octanol and acetate buffer (pH 4.2) + octanol. Each data point represents the mean of four replicates with error bars denoting standard deviations

**Fig. 5:**
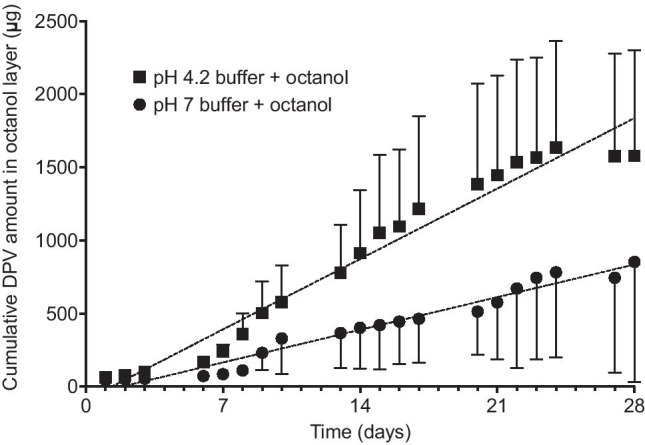
Mean cumulative amount of DPV measured in the octanol phase of the buffer/octanol system for phosphate buffer (pH 7) + octanol and acetate buffer (pH 4.2) + octanol. Each data point represents the mean of four replicates with error bars denoting standard deviations

The mean cumulative amount of DPV released from rings into the aqueous buffer and octanol components of the two-phase release media are presented in Figs. 4 and 5, respectively. The quantity of DPV present in the 100 mL buffer components (i) was generally less than 15 µg (consistent with its extremely poor aqueous solubility, Table 1), (ii) was dependent upon buffer pH, with greater release at pH 4.2 compared to 7.0 (consistent with DPV’s pKa value and ionisation behaviour, Table 1), and (iii) showed much greater variability compared with the IPA/water release data. That the weakly basic DPV molecule exists predominately in its protonated form (DPV-H^+^) at normal healthy acidic vaginal pH may explain its relatively low systemic absorption following vaginal administration [51–53]. A small number of previous studies have sought to determine whether the principles of pH partition theory–which have been widely considered and are well established for intestinal drug absorption–also apply to ionisable drugs administered vaginally [49, 54–58]. It would be interesting to assess whether vaginal pH impacts DPV systemic absorption in the clinic, particularly given the very high prevalence of bacterial vaginosis among women in sub-Saharan Africa, where the DPV ring is intended for use [59–61].

The mean cumulative amount of DPV measured in the octanol phases of the two biphasic release media increased with time (Fig. 5), in line with the expectation that hydrophobic DPV molecules (log *P* 5.35, Table 1) dissolved in the aqueous buffer phase will preferentially partition into the octanol layer. Larger quantities of DPV were measured in the octanol/acetate buffer (pH 4.2) system, consistent with the higher concentrations of DPV measured in the acetate buffer (Fig. 3). Further, the much higher quantities measured in the octanol phase compared to the aqueous buffers are broadly consistent with pharmacokinetic concentrations measured in vivo, where the octanol can be considered to broadly represent the combined vaginal tissue + systemic compartments and the aqueous buffer the vaginal fluid. We appreciate that the buffer volumes used in these in vitro models are much larger than vaginal fluid volumes in vivo [24]. Nonetheless, this simple octanol/buffer system represents a first attempt at better understanding and modelling drug concentrations in the various biological compartments.

The data for DPV quantity measured in the octanol phase are highly variable (see large errors bars in Fig. 5). The mean rates of DPV accumulation in the octanol phase for each of the two systems are presented in Table 3 along with 95% confidence intervals, *R*^2^ values, and the mean cumulative amount released over 28 days. Overall, the total amount and rate of DPV release into the acetate buffer/octanol system is approximately double that measured with the phosphate buffer/octanol system.

**Table 3:** Mean cumulative amount released on day 28, cumulative release rate equation gradients and intercepts with 95% confidence intervals in brackets, and coefficients of determination for DPV release into two different biphasic buffer/octanol systems

Release media	Mean cumulative amount released on day 28 (mg) ± SD	Slope for octanol data (µg/day)	Intercept	*R*^2^ value
pH 7 buffer + octanol	0.85 ± 0.82	31.88 (22.89, 40.88)	−57.15 (−205.2, 90.91)	0.3898
pH 4.2 buffer + octanol	1.58 ± 0.72	68.81 (56.67, 80.95)	−89.70 (−289.6, 110.2)	0.6202
pH 7 buffer + octanol − ring 4	0.44 ± 0.11	17.77 (14.81, 20.73)	9.91 (−38.84, 58.66)	0.7134
pH 4.2 buffer + octanol − ring 5, 8	0.98 ± 0.14	40.94 (38.19, 43.69)	−26.24 (−71.52, 19.03)	0.9598

The high variability associated with the DPV quantities measured in octanol and the low *R*^2^ values (Table 3) require further comment. Plots of individual ring data (Supplementary Information Fig. S3A, B) show that for the pH 7 buffer/octanol system, three rings follow each other quite closely over the 28 days, while one ring diverges from day 10 onwards (ring 4, Fig. S3A). In the plot of the pH 4 buffer/octanol system, two rings follow each other closely while the other two diverge from day 8 onwards (rings 5 and 8, Supplementary Information Fig. S3B). The large and anomalous increases in release with rings 4, 5, and 8 compared to other rings are likely due to inadvertent ring contact with the octanol phase (for example during sampling), and highlight a particular challenge with use of a biphasic release medium. The adjusted release rates with these anomalous data removed (Table 3) are approximately half of the original values. However, the data still support a faster rate of DPV release with the octanol/acetate buffer system, in line with expectations of increased tissue/systemic absorption at lower vaginal pH.

The total amount of DPV released from the 25 mg DPV ring following 28-day in vivo use is ~4 mg, as determined by extracting and quantifying residual DPV in rings following clinical use [62–64]. Clearly, the amounts of DPV released in vitro using the octanol/water media (~1 mg measured in the octanol phase) are lower than those following in vivo use, despite the much larger volumes of buffer used (100 mL) relative to those of vaginal fluid (~2 mL). Possible reasons for this discrepancy include the following: (i) vaginal fluid contains various small organic molecules and proteins that likely enhance solubility of poorly water-soluble drugs [24, 65], (ii) direct diffusion of drug from the ring surface into the adjacent tissue (or, at least, drug diffusion through a much thinner aqueous diffusion (hydrostatic) layer, (iii) different fluid dynamics and turnover rates for vaginal fluid compared with the aqueous buffer. Although simple buffers and SVF are widely used as single-phase media for in vitro release testing of vaginal rings containing poorly water-soluble drugs, the addition of surfactants is often required to enhance drug solubility and obtain meaningful release data. In this study, we opted not to add surfactants to the buffer solutions since we anticipated that concomitant use of octanol (albeit as a second immiscible phase) would adequately compensate. Assuming the measured release data scale linearly, an aqueous fluid volume of ~ 350 mL should provide the necessary release rates to match the amounts of DPV released in vivo.

Potential strategies to reduce the variability seen in DPV release into the two-phase system include the following: (i) decreasing the orbital shaking speed of the incubator to reduce the potential for ring contact with the octanol phase, (ii) use of a magnetic stirring system rather than a shaking incubator system, (iii) sampling consistently using a needle and syringe (rather than a micropipette) throughout the experiment, and (iv) using a larger aqueous volume. Initially, we used a micropipette to sample both octanol and buffer phases. From day 14, a needle and syringe was used to reduce potential contamination with octanol when sampling the underlying aqueous layer. Indeed, octanol droplets were observed in some aqueous layer samples (Supplementary Information, Fig. S4), which may account for some of the variability in the data.

It would also be interesting to assess if the ratios of drug in the biphasic octanol/buffer system correspond to the ratios observed in tissue/vaginal fluid or tissue/plasma. A cursory review of published pharmacokinetic data suggests no obvious correlations. In general, much higher concentrations of DPV are observed in vaginal fluids, suggesting that tissue/vaginal fluid ratios, for example, are opposite to what we observe in this in vitro study. Ideally, a system that could more closely replicate the composition, volume, and rate of turnover of vaginal fluid would be preferable.

## Conclusions

The release of DPV from a 25 mg matrix ring transitioned from a partition-controlled to permeation-controlled mechanism upon increasing the IPA fraction in an IPA/water mixture from 10 to 20%. Use of a 20/80 IPA/water mixture provided release of approximately 3.5 mg DPV over 28 days which is close to the ~4 mg released over 28 days in vivo. By comparison, use of a 30/70 IPA/water mixture gave release of 8 mg DPV. Increases in release rate upon further addition of IPA to the release medium suggest that the transition between partition-controlled and matrix-controlled release is relatively broad in this system. Using the Peppas equation, true Fickian release was not observed even at 50/50 IPA/water, despite this representing sink conditions according to measured DPV solubility value and daily release concentrations. Use of a biphasic aqueous-octanol release medium provided release of up to ~1 mg of DPV over 28 days. Clear differences in the amount of DPV accumulating in the octanol phase were measured between release into a phosphate buffer (pH 7) and acetate buffer (pH 4.2) aqueous phase. This is in line with the increased solubility of the ionised form of DPV predominating at pH 4.2.

## Supplementary information

Below is the link to the electronic supplementary material.

Supplementary file1 (DOCX 390 MB)

## Data Availability

All authors agree that any materials and data that are reasonably requested by others will be made available for non-commercial purposes.

## References

[1] Miranda M, Pais AACC, Cardoso C, Vitorino C. aQbD as a platform for IVRT method development – a regulatory oriented approach. Int J Pharm [Internet]. Elsevier B.V; 2019;572:118695. Available from: 10.1016/j.ijpharm.2019.118695. 10.1016/j.ijpharm.2019.118695 31536762

[2] Bao Q, Zou Y, Wang Y, Choi S, Burgess DJ. Impact of product design parameters on in vitro release from intrauterine systems. Int J Pharm [Internet]. Elsevier; 2020;578:119135. Available from: 10.1016/j.ijpharm.2020.119135. 10.1016/j.ijpharm.2020.119135 PMC8220931 32057890

[3] Bao Q, Zou Y, Wang Y, Kozak D, Choi S, Burgess DJ. Drug release testing of long-acting intrauterine systems. J Control Release [Internet]. Elsevier; 2019;316:349–58. Available from: 10.1016/j.jconrel.2019.11.015. 10.1016/j.jconrel.2019.11.015 PMC8205262 31733294

[4] Boyd P, Variano B, Spence P, McCoy CF, Murphy DJ, Dallal Bashi YH, et al. In vitro release testing methods for drug-releasing vaginal rings. J Control Release [Internet]. Elsevier; 2019;313:54–69. Available from: 10.1016/j.jconrel.2019.10.015. 10.1016/j.jconrel.2019.10.015 31626862

[5] Tietz K, Klein S. In vitro methods for evaluating drug release of vaginal ring formulations–a critical review. Pharmaceutics. 2019;11. 10.3390/pharmaceutics11100538 PMC6836189 31623277

[6] Shen J, Burgess DJ. In vitro-in vivo correlation for complex non-oral drug products: where do we stand? J Control Release [Internet]. Elsevier B.V.; 2015;219:644–51. Available from: 10.1016/j.jconrel.2015.09.052. 10.1016/j.jconrel.2015.09.052 PMC4739855 26419305

[7] Externbrink A, Clark MR, Friend DR, Klein S. European Journal of Pharmaceutics and Biopharmaceutics Investigating the feasibility of temperature-controlled accelerated drug release testing for an intravaginal ring. Eur J Pharm Biopharm [Internet]. Elsevier B.V.; 2013;85:966–73. Available from: 10.1016/j.ejpb.2013.06.004. 10.1016/j.ejpb.2013.06.004 23791685

[8] Abend A, Curran D, Kuiper J, Lu X, Li H, Hermans A, et al. Dissolution testing in drug product development: workshop summary report. AAPS J [Internet]. 2019;21:21. Available from: 10.1208/s12248-018-0288-4. 10.1208/s12248-018-0288-4 30690680

[9] Grady H, Elder D, Webster GK, Mao Y, Lin Y, Flanagan T, et al. Industry’s view on using quality control, biorelevant, and clinically relevant dissolution tests for pharmaceutical development, registration, and commercialization. J Pharm Sci [Internet]. Elsevier Ltd; 2018;107:34–41. Available from: 10.1016/j.xphs.2017.10.019. 10.1016/j.xphs.2017.10.019 29074376

[10] Suarez-Sharp S, Cohen M, Kesisoglou F, Abend A, Marroum P, Delvadia P (2018). Applications of clinically relevant dissolution testing: workshop summary report. AAPS J The AAPS Journal.

[11] Marroum PJ. Clinically relevant dissolution methods and specifications. Am Pharm Rev. 2012;15.

[12] Heimbach T, Suarez-Sharp S, Kakhi M, Holmstock N, Olivares-Morales A, Pepin X (2019). Dissolution and translational modeling strategies toward establishing an in vitro-in vivo link–a workshop summary report. AAPS J The AAPS Journal.

[13] Mann J, Dressman J, Rosenblatt K, Ashworth L, Muenster U, Frank K (2017). Validation of dissolution testing with biorelevant media: an OrBiTo study. Mol Pharm.

[14] McBride JW, Boyd P, Dias N, Cameron D, Offord RE, Hartley O, et al. Vaginal rings with exposed cores for sustained delivery of the HIV CCR5 inhibitor 5P12-RANTES. J Control Release [Internet]. 2019;298:1–11. Available from: https://linkinghub.elsevier.com/retrieve/pii/S016836591930077X. 10.1016/j.jconrel.2019.02.003 PMC6414755 30731150

[15] Malcolm RK, Boyd PJ, McCoy CF, Murphy DJ. Microbicide vaginal rings: technological challenges and clinical development. Adv Drug Deliv Rev [Internet]. Elsevier B.V.; 2016;103:33–56. Available from: 10.1016/j.addr.2016.01.015. 10.1016/j.addr.2016.01.015 26829289

[16] Murphy DJ, McCoy CF, Boyd P, Derrick T, Spence P, Devlin B, et al. Drug stability and product performance characteristics of a dapivirine-releasing vaginal ring under simulated real-world conditions. Int J Pharm [Internet]. Elsevier; 2019;565:351–7. Available from: 10.1016/j.ijpharm.2019.05.027. 10.1016/j.ijpharm.2019.05.027 31085254

[17] McCoy CF, Murphy DJ, Boyd P, Derrick T, Spence P, Devlin B, et al. Packing polymorphism of dapivirine and its impact on the performance of a dapivirine-releasing silicone elastomer vaginal ring. J Pharm Sci [Internet]. 2017;106:2015–25. Available from: http://linkinghub.elsevier.com/retrieve/pii/S0022354917302617. 10.1016/j.xphs.2017.04.026 28456732

[18] Ugaonkar SR, Wesenberg A, Wilk J, Seidor S, Mizenina O, Kizima L, et al. A novel intravaginal ring to prevent HIV-1, HSV-2, HPV, and unintended pregnancy. J Control Release [Internet]. Elsevier B.V.; 2015;213:57–68. Available from: http://www.sciencedirect.com/science/article/pii/S0168365915006252. 10.1016/j.jconrel.2015.06.018 PMC4683108 26091920

[19] Su JT, Teller RS, Srinivasan P, Zhang J, Martin A, Sung S, et al. A dose ranging pharmacokinetic evaluation of IQP-0528 released from intravaginal rings in non-human primates. Pharm Res [Internet]. Pharm Res. 2017;34:2163–71. Available from: http://www.ncbi.nlm.nih.gov/pubmed/28770490. 10.1007/s11095-017-2224-1 PMC7036280 28770490

[20] Murphy DJ, Desjardins D, Boyd P, Dereuddre-bosquet N, Stimmer L, Caldwell A, et al. Impact of ring size and drug loading on the pharmacokinetics of a combination dapivirine-darunavir vaginal ring in cynomolgus macaques. Int J Pharm [Internet]. Elsevier; 2018;550:300–8. Available from: 10.1016/j.ijpharm.2018.08.051. 10.1016/j.ijpharm.2018.08.051 30153490

[21] Johnson TJ, Srinivasan P, Albright TH, Watson-Buckheit K, Rabe L, Martin A (2012). Safe and sustained vaginal delivery of pyrimidinedione HIV-1 inhibitors from polyurethane intravaginal rings. Antimicrob Agents Chemother.

[22] Shohin IE, Grebenkin DY, Malashenko EA, Stanishevskii YM, Ramenskaya GV (2016). A brief review of the FDA dissolution methods database. Dissolution Technol.

[23] Bélec L, Meillet D, Levy M, Georges A, Tevi-Benissan C, Pillot J (1995). Dilution assessment of cervicovaginal secretions obtained by vaginal washing for immunological assays. Clin Diagn Lab Immunol.

[24] Owen DH, Katz DF (1999). A vaginal fluid simulant. Contraception.

[25] Mitchell C, Paul K, Agnew K, Gaussman R, Coombs RW, Hitti J. Estimating volume of cervicovaginal secretions in cervicovaginal lavage fluid collected for measurement of genital HIV-1 RNA levels in women box. 2011;49:735–6. 10.1128/JCM.00991-10 PMC3043490 21106793

[26] Donoso MB, Serra R, Rice GE, Gana MT, Rojas C, Khoury M, et al. Normality ranges of menstrual fluid volume during reproductive life using direct quantification of menses with vaginal cups. Gynecol Obstet Invest [Internet]. 2019;1–6. Available from: http://www.ncbi.nlm.nih.gov/pubmed/30712040. 10.1159/000496608 30712040

[27] Patton DL, Thwin SS, Meier A, Hooton TM, Stapleton AE, Eschenbach DA. Epithelial cell layer thickness and immune cell populations in the normal human vagina at different stages of the menstrual cycle. Am J Obstet Gynecol. 2000;183:967–73. 10.1067/mob.2000.108857 11035348

[28] Katz DF, Yuan A, Gao Y. Vaginal drug distribution modeling. Adv Drug Deliv Rev [Internet]. Elsevier B.V.; 2015; Available from: http://linkinghub.elsevier.com/retrieve/pii/S0169409X15000812. 10.1016/j.addr.2015.04.017 PMC4600641 25933938

[29] Pendergrass PB, Belovicz MW, Reeves CA (2003). Surface area of the human vagina as measured from vinyl polysiloxane casts. Gynecol Obstet Invest.

[30] Tietz K, Klein S. Simulated genital tract fluids and their applicability in drug release/dissolution testing of vaginal dosage forms. Dissolution Technol [Internet]. 2018;25:40–51. Available from: http://www.dissolutiontech.com/issues/201808/DT201808_A04.pdf.

[31] Rastogi R, Su J, Mahalingam A, Clark J, Sung S, Hope T, et al. Engineering and characterization of simplified vaginal and seminal fluid simulants. Contraception [Internet]. Elsevier Inc.; 2016;93:337–46. Available from: 10.1016/j.contraception.2015.11.008. 10.1016/j.contraception.2015.11.008 PMC4863460 26585883

[32] Teller RS, Malaspina DC, Rastogi R, Clark JT, Szleifer I, Kiser PF. Controlling the hydration rate of a hydrophilic matrix in the core of an intravaginal ring determines antiretroviral release. J Control Release [Internet]. Elsevier B.V.; 2016;224:176–83. Available from: 10.1016/j.jconrel.2015.12.035. 10.1016/j.jconrel.2015.12.035 PMC4783791 26723526

[33] Moss JA, Butkyavichene I, Churchman SA, Gunawardana M, Fanter R, Miller CS (2016). Combination pod-intravaginal ring delivers antiretroviral agents for HIV prophylaxis: pharmacokinetic evaluation in an ovine model. Antimicrob Agents Chemother.

[34] Baeten JM, Palanee-Phillips T, Brown ER, Schwartz K, Soto-Torres LE, Govender V, et al. Use of a vaginal ring containing dapivirine for HIV-1 prevention in women. N Engl J Med [Internet]. 2016;375:2121–32. Available from: http://www.ncbi.nlm.nih.gov/pubmed/26900902. 10.1056/NEJMoa1506110 PMC4993693 26900902

[35] Nel A, van Niekerk N, Kapiga S, Bekker L-G, Gama C, Gill K, et al. Safety and efficacy of a dapivirine vaginal ring for HIV prevention in women. N Engl J Med [Internet]. 2016;375:2133–43. Available from:10.1056/NEJMoa1602046. 10.1056/NEJMoa1602046 27959766

[36] Spence P, Nel A, van Niekerk N, Derrick T, Wilder S, Devlin B. Post-use assay of vaginal rings (VRs) as a potential measure of clinical trial adherence. J Pharm Biomed Anal [Internet]. Elsevier B.V.; 2016;125:94–100. Available from: 10.1016/j.jpba.2016.03.023. 10.1016/j.jpba.2016.03.023 PMC4873601 27016673

[37] Pestieau A, Evrard B. In vitro biphasic dissolution tests and their suitability for establishing in vitro-in vivo correlations: a historical review. Eur J Pharm Sci [Internet]. Elsevier B.V.; 2017;102:203–19. Available from: 10.1016/j.ejps.2017.03.019. 10.1016/j.ejps.2017.03.019 28315463

[38] Yasuda M. Dissociation constants of some carboxylic acids in mixed aqueous solvents. Bull Chem Soc Jpn [Internet]. 1959;32:429–32. Available from: 10.1246/bcsj.32.429.

[39] Völgyi G, Ruiz R, Box K, Comer J, Bosch E, Takács-Novák K (2007). Potentiometric and spectrophotometric pKa determination of water-insoluble compounds: validation study in a new cosolvent system. Anal Chim Acta.

[40] Higuchi T. Mechanism of sustained-action medication. Theoretical analysis of rate of release of solid drugs dispersed in solid matrices. J Pharm Sci. 1963;52:1145–9. 10.1002/jps.2600521210 14088963

[41] Paul DR. Elaborations on the Higuchi model for drug delivery. Int J Pharm [Internet]. Elsevier B.V.; 2011;418:13–7. Available from: 10.1016/j.ijpharm.2010.10.037. 10.1016/j.ijpharm.2010.10.037 21034800

[42] Peppas NA. 1. Commentary on an exponential model for the analysis of drug delivery: original research article: a simple equation for description of solute release: I II. Fickian and non-Fickian release from non-swellable devices in the form of slabs, spheres, cylind. J Control Release [Internet]. Elsevier B.V.; 2014;190:31–2. Available from: 10.1016/S0168-3659(14)00482-9. 25356469

[43] Ritger PL, Peppas NA. A simple equation for description of solute release I. Fickian and non-fickian release from non-swellable devices in the form of slabs, spheres, cylinders or discs. J Control Release [Internet]. 1987;5:23–36. Available from: https://linkinghub.elsevier.com/retrieve/pii/0168365987900344. 25356469

[44] Frenkel YV, Clark AD, Das K, Wang YH, Lewi PJ, Janssen PA (2005). Concentration and pH dependent aggregation of hydrophobic drug molecules and relevance to oral bioavailability. J Med Chem..

[45] Völgyi G, Baka E, Box KJ, Comer JE, Takács-Novák K. Study of pH-dependent solubility of organic bases. Revisit of Henderson-Hasselbalch relationship. Anal Chim Acta [Internet]. 2010;673:40–6. Available from: http://www.ncbi.nlm.nih.gov/pubmed/20630176. 10.1016/j.aca.2010.05.022 20630176

[46] Baka E, Comer JE, Takács-Novák K. Study of equilibrium solubility measurement by saturation shake-flask method using hydrochlorothiazide as model compound. J Pharm Biomed Anal [Internet]. 2008;46:335–41. Available from: http://www.ncbi.nlm.nih.gov/pubmed/18055153. 10.1016/j.jpba.2007.10.030 18055153

[47] Miller JM, Beig A, Carr RA, Webster GK, Dahan A (2012). The solubility-permeability interplay when using cosolvents for solubilization: revising the way we use solubility-enabling formulations. Mol Pharm.

[48] Siepmann J, Siepmann F. Sink conditions do not guarantee the absence of saturation effects. Int J Pharm [Internet]. Elsevier; 2020;577:119009. Available from: 10.1016/j.ijpharm.2019.119009. 10.1016/j.ijpharm.2019.119009 31917299

[49] Fetherston SM, Geer L, Veazey RS, Goldman L, Murphy DJ, Ketas TJ, et al. Partial protection against multiple RT-SHIV162P3 vaginal challenge of rhesus macaques by a silicone elastomer vaginal ring releasing the NNRTI MC1220. J Antimicrob Chemother [Internet]. 2013;68:394–403. Available from: 10.1093/jac/dks415. 10.1093/jac/dks415 PMC3543122 23109186

[50] Chien YW, Lambert HJ. Controlled drug release from polymeric delivery devices II: differentiation between partition-controlled and matrix-controlled drug release mechanisms. J Pharm Sci [Internet]. 1974;63:515–9. Available from: https://linkinghub.elsevier.com/retrieve/pii/S0022354915415795. 10.1002/jps.2600630405 4828696

[51] Nel A, Smythe S, Young K, Malcolm K, McCoy C, Rosenberg Z, et al. Safety and pharmacokinetics of dapivirine delivery from matrix and reservoir intravaginal rings to HIV-negative women. J Acquir Immune Defic Syndr [Internet]. United States; 2009;51:416–23. Available from: http://www.ncbi.nlm.nih.gov/pubmed/19623693. 10.1097/qai.0b013e3181acb536 19623693

[52] Nel A, Haazen W, Nuttall J, Romano J, Rosenberg Z, Van Niekerk N. A safety and pharmacokinetic trial assessing delivery of dapivirine from a vaginal ring in healthy women. Aids [Internet]. 2014;28:1479–87. Available from: http://www.ncbi.nlm.nih.gov/pubmed/24901365. 10.1097/QAD.0000000000000280 24901365

[53] Nel AM, Haazen W, Nuttall JP, Romano JW, Mesquita PMM, Herold BC, et al. Pharmacokinetics and safety assessment of anti-HIV dapivirine vaginal microbicide rings with multiple dosing. J AIDS Clin Res [Internet]. 2014;05. Available from: https://www.omicsonline.org/open-access/pharmacokinetics-and-safety-assessment-of-antihiv-dapivirine-vaginal-microbicide-rings-with-multiple-dosing-2155-6113.1000355.php?aid=32193.

[54] Gunalp S, Bildirici I (2000). The effect of vaginal pH on the efficacy of vaginal misoprostol for induction of labor. Acta Obstet Gynecol Scand.

[55] van Eyk AD, van Der Bijl P, Moll LM (2008). Physicochemical characteristics of molecules and their diffusion across human vaginal mucosa. Eur J Inflamm.

[56] Van Der Bijl P, Penkler L, Van Eyk AD (2000). Permeation of sumatriptan through human vaginal and buccal mucosa. Headache.

[57] Ramsey PS, Ogburn PL, Harris DY, Heise RH, Ramin KD (2000). Effect of vaginal pH on efficacy of misoprostol for cervical ripening and labor induction. Am J Obstet Gynecol.

[58] Singh K, Fong YF, Prasad RNV, Dong R (1999). Does an acidic medium enhance the efficacy of vaginal misoprostol for pre-abortion cervical priming?.. Hum Reprod.

[59] Torrone EA, Morrison CS, Chen PL, Kwok C, Francis SC, Hayes RJ, et al. Prevalence of sexually transmitted infections and bacterial vaginosis among women in sub-Saharan Africa: an individual participant data meta-analysis of 18 HIV prevention studies. PLoS Med. 2018. 10.1371/journal.pmed.1002511 PMC5828349 29485986

[60] Chico RM, Mayaud P, Ariti C, Mabey D, Ronsmans C, Chandramohan D (2012). Prevalence of malaria and sexually transmitted and reproductive tract infections in pregnancy in sub-Saharan Africa: a systematic review. JAMA - J Am Med Assoc.

[61] Francis SC, Looker C, Vandepitte J, Bukenya J, Mayanja Y, Nakubulwa S (2016). Bacterial vaginosis among women at high risk for HIV in Uganda: high rate of recurrent diagnosis despite treatment. Sex Transm Infect.

[62] Baeten JM, Palanee-Phillips T, Mgodi NM, Mayo AJ, Szydlo DW, Ramjee G (2021). Safety, uptake, and use of a dapivirine vaginal ring for HIV-1 prevention in African women (HOPE): an open-label, extension study. Lancet HIV.

[63] Nel A, Bekker L-G, Bukusi E, Hellstrӧm E, Kotze P, Louw C, et al. Safety, Acceptability and adherence of dapivirine vaginal ring in a microbicide clinical trial conducted in multiple countries in Sub-Saharan Africa. PLoS One [Internet]. 2016;11:e0147743. Available from: http://www.pubmedcentral.nih.gov/articlerender.fcgi?artid=4786336&tool=pmcentrez&rendertype=abstract. 10.1371/journal.pone.0147743 PMC4786336 26963505

[64] Spence P, Nel A, van Niekerk N, Derrick T, Wilder S, Devlin B. Post-use assay of vaginal rings (VRs) as a potential measure of clinical trial adherence. J Pharm Biomed Anal [Internet]. Elsevier B.V.; 2016;125:94–100. Available from: http://www.sciencedirect.com/science/article/pii/S0731708516301418. 10.1016/j.jpba.2016.03.023 PMC4873601 27016673

[65] Kim JY, Kim S, Papp M, Park K, Pinal R. Hydrotropic solubilization of poorly water-soluble drugs. J Pharm Sci [Internet]. Elsevier Masson SAS; 2010;99:3953–65. Available from: 10.1002/jps.22241. 10.1002/jps.22241 20607808

