# Supplementary material for: Refining the in vitro release test method for a dapivirine-releasing vaginal ring to match in vivo performance
Source: Drug Deliv Transl Res. 2021 Oct 21;13(8):2072–82. doi: 10.1007/s13346-021-01081-7 (PMC10315348; doi:10.1007/s13346-021-01081-7)
Supplement: Supplementary file 1 — Supplementary file1 (DOCX 390 MB) [file 13346_2021_1081_MOESM1_ESM.docx]

**Supplementary Information**

*DPV Calibration curve*

DPV standards were prepared at between 0.2 and 100 µg/mL in a mixture of IPA/water in three independent replicates. The calibration curve was weighted by the amount injected and the summary output is displayed in Table S1, the curve is displayed in Figure S1 below.

Table S1. Calibration curve values for the quantification of DPV

|  | Estimate | Std. Error |
| --- | --- | --- |
| Intercept | -454.77 | 280.58 |
| DPV amount | 4132.32 | 31.03 |

Adjusted R-squared: 0.998.

Figure S1. Weighted calibration curve for the quantification of DPV.

*Dapivirine pKa determination*

Dapivirine exhibits a single pKa in the pH range 2–12. This was determined by titration in a mixed solvent system consisting of acetonitrile, dioxane and methanol in potassium chloride and extrapolated to zero co-solvent to derive the aqueous value using the methods of Yasuda-Shedlovsky. This value is lower than a previously published pKa value for dapivirine, but no methodology was given for the previously published value so it is not possible to compare them.

Figure S2. Yasuda-Shedlovsky extrapolation for the pKa determination of dapivirine in a fully aqueous system.

Figure S3. DPV accumulation by individual rings in the octanol layer of an (A) phosphate buffer (pH 7) - octanol two phase system, (B) acetate buffer (pH 4.2) – octanol two phase system.


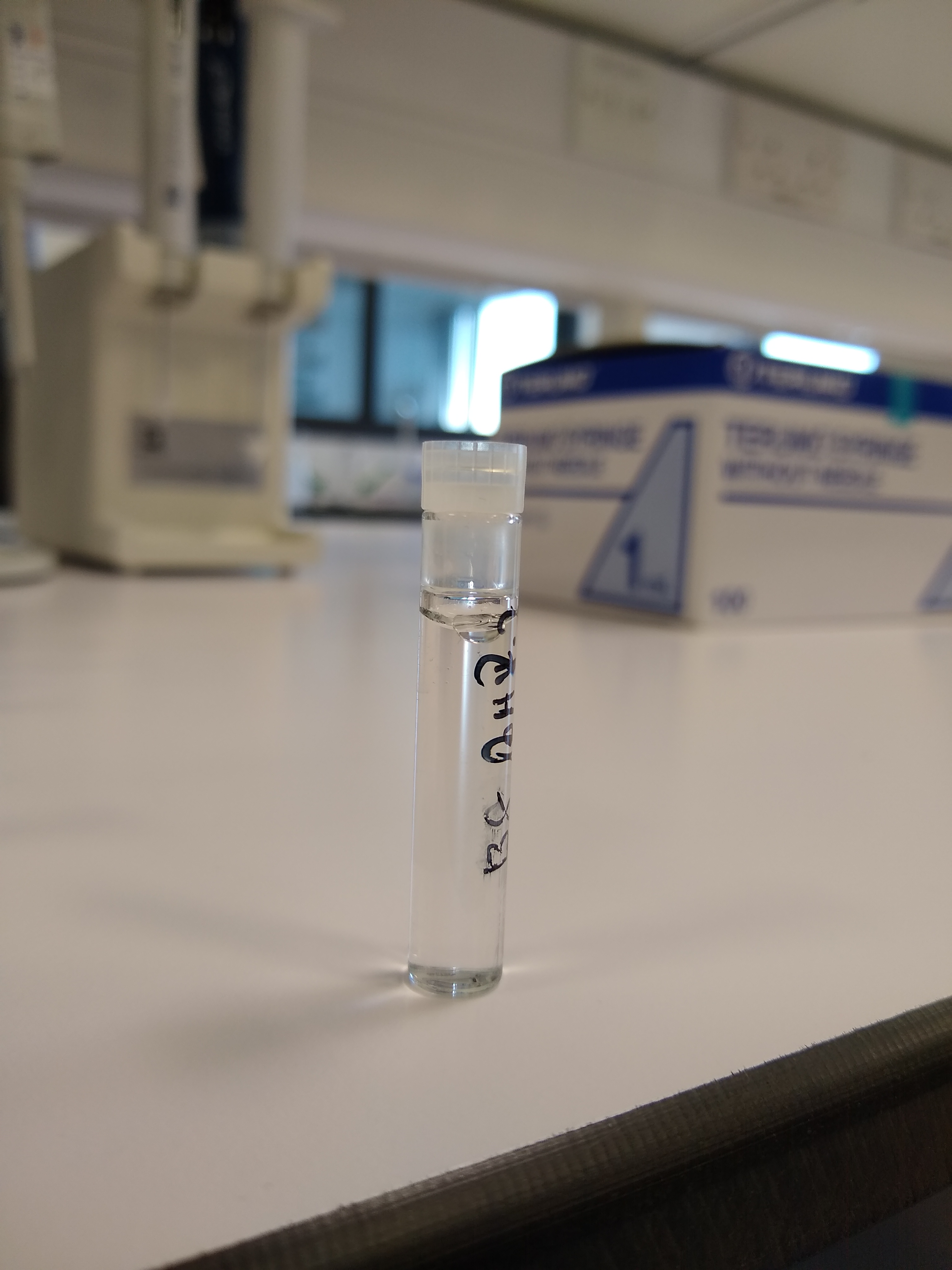

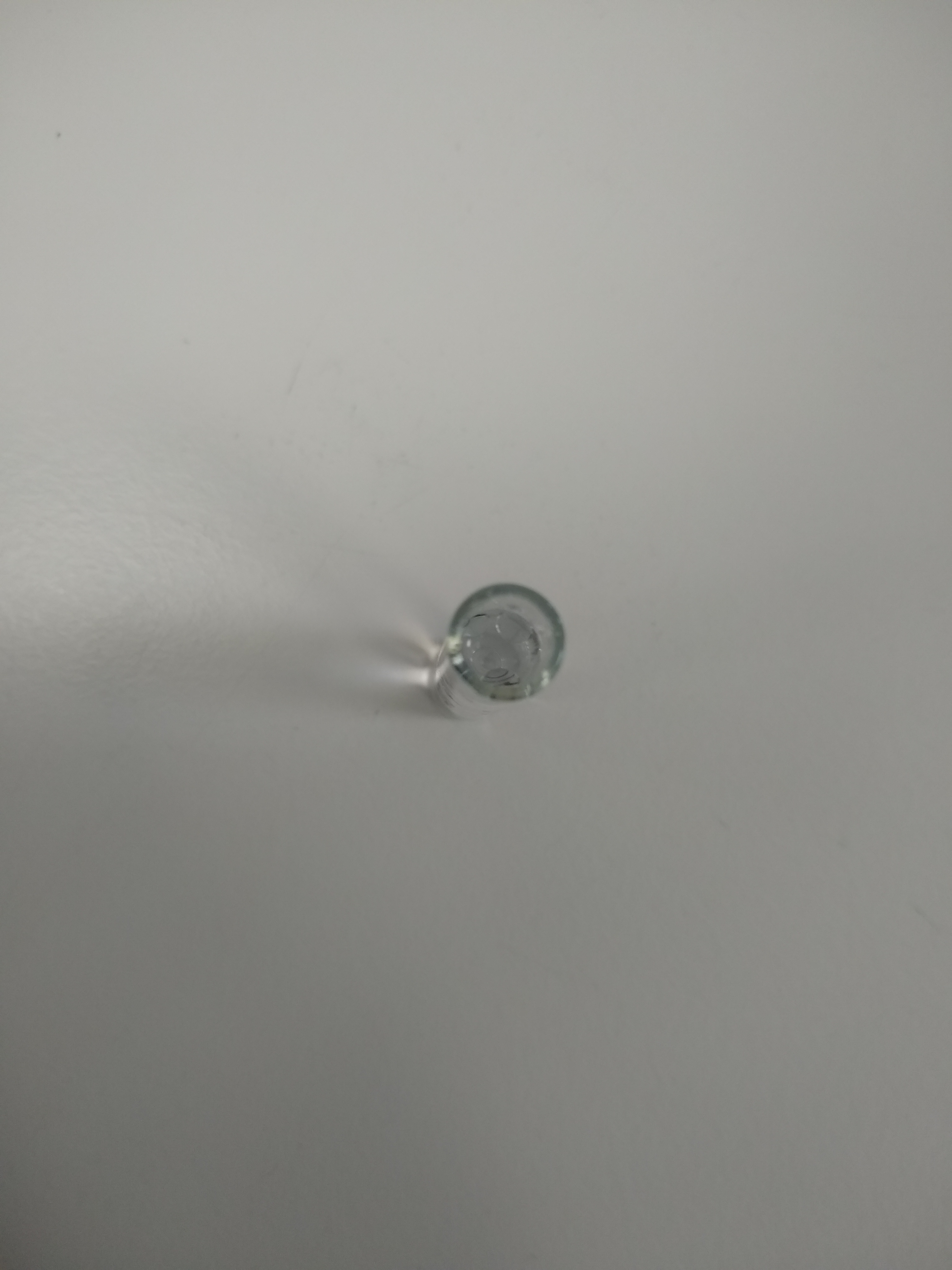


Figure S4. Octanol droplets observed in the buffer samples taken during the two phase release experiment.
